# Faecal microbiota transplantation: looking beyond clostridium difficile infection at inflammatory bowel disease 

**Published:** 2018

**Authors:** Krish Patel, Amee Patel, David Hawes, Janki Shah, Krishna Shah

**Affiliations:** 1 *Barts and the London School of Medicine and Dentistry, Queen Mary University of London, UK*; 2 *GKT School of Medical Education, King’s College London, UK *; 3 *Plymouth University Peninsula Schools of Medicine and Dentistry, UK*; 4 *Department of Medicine, Imperial College School of Medicine, UK*

**Keywords:** Inflammatory bowel disease, Ulcerative colitis, Crohn’s disease, Faecal microbiota transplantation

## Abstract

Gastrointestinal (GI) microbiota are known to play paramount role in inflammatory bowel disease (IBD). Innovative sequencing methods have radically expanded our ability to analyze the intestinal microbiome. However, alterations of the GI microbiome in IBD have not yet been fully evaluated. Irregular colonization of the gut has been implicated in chronic intestinal inflammation. Faecal microbiota transplantation (FMT) is a procedure which aims to restore microbial disturbances to the individual’s gut microbiome. The success of FMT in Clostridium difficile infection (CDI) has inspired studies to explore transplantation in other conditions such as IBD. Ulcerative colitis (UC) and Crohn’s disease (CD), the two principal manifestations of IBD, are emerging as a worldwide epidemic and are multifactorial in aetiology. There have been various case series in the past looking at the use of FMT in IBD, with a large number of them focusing on UC; however, two new randomized controlled trials shed up-to-date light on the complex interactions between the GI microbiome and patients. Regardless of these new studies, much more remains unknown about the efficacy and safety profile of FMT in IBD, ultimately casting a shadow over its use as a therapeutic intervention in conditions other than CDI. Further researches are necessary to fully evaluate the role of FMT as a management option in IBD. In this review, we discuss and summarize the functions of FMT in IBD, and the relationship between IBD and the GI microbial variations present.

## Introduction

 Understanding the role of gut microbiota in humans provides a fundamental insight into bowel disease. The advent of novel sequencing methods has radically increased our capability to analyse the human microbiome. It is now estimated that of the 10–100 trillion microbes present in a healthy human gastrointestinal (GI) tract, the majority are present in the colon. The GI microbiome is dominated by two phyla: Firmicutes and Bacteroidetes (1). As the composition of gut microbiota is implicated in pathology, there has been a surge of interest in developing strategies that alter the microbiome.

Faecal microbiota transplantation (FMT) is a therapeutic intervention used to transfer faecal samples from healthy donors into patients. The aim of the procedure is to re-colonise the individual’s gut microbiome using the colonic bacteria of a healthy donor to attenuate pathogenic processes. The microbial element of stool samples is separated through laboratory techniques, with a viability to cryogenically freeze the sample and thus intensify the efficacy (2). This is done by using an enema, colonoscopy, or a variety of options through the upper GI tract. The primary clinical use of FMT currently, is for the treatment of recurrent *Clostridium difficile* infection (rCDI) (3). Recently, there has been an increase of interest in utilising FMT for other disorders, such as inflammatory bowel disease (IBD), irritable bowel syndrome (IBS) and metabolic disorders including obesity and type 2 diabetes mellitus (T2DM) (4-6).

IBD is a cluster of inflammatory autoimmune diseases that primarily affect the colon and small intestine. Ulcerative colitis (UC) and Crohn’s disease (CD) are the predominant manifestations of IBD, and are emerging as a worldwide epidemic (7). The true pathophysiology of IBD remains unknown; however, an alteration in the GI microbiome in genetically susceptible individuals, environmental factors, and a modified immune system have all been predicted to have a part in its pathogenesis. The current therapeutic interventions used in IBD can regulate the immune system; however, are used with restriction due to their toxic side effects and lack of effectiveness (8). Patients with IBD display a reduced regulation of the GI microbiome, resulting in gut microbial dysbiosis (9). Irregular colonisation of the gut leads to a dysregulation in signalling between gut microbiota and the immune system (9). This event has been implicated in chronic intestinal inflammation (10, 11). In clinically active UC, administration of a broad-spectrum antibiotic leds to a decrease of stimulators of inflammation including in interleukin-8 (IL-8) (12). Thus, implicating intraluminal microbiota in disease pathogenesis by causing damage to the mucosa via immunoinflammatory responses grounded on this pathophysiological notion, utilising FMT in the management of IBD appears rational.

**The Gut microbiome in inflammatory bowel disease**

*Escherichia coli* (*E. coli*), *Lactobacillus casei *(*L. case*i) and *Faecalibacterium prausnitzii* (*F. prausnitzii*) are commensal non-pathogenic bacteria which have contradictory effects on key proinflammatory mediators that are responsible for the development of inflammatory infiltrates in the intestine (13). In patients with IBD, *E. coli *was found to powerfully upregulate proinflammatory cytokines and chemokines (tumour necrosis factor-alpha (TNF-α), interferon-gamma (IFN-γ), IL-6, IL-8, IL-17F, IL-23p19, CXCL1 and CXCL2) (13,14). The ensuing cascade triggers a stimulation of matrix metalloproteinases (MMPs) and ultimately leads to epithelium damage (15). Conversely, in other studies, Faecalibacterium, Lactobacillus and Bifidobacterium are found to offset the immunoinflammatory effects of *E. coli *(13). The colonization of Firmicutes, Bacteroidetes and the overall GI microbiome diversity are all reduced in IBD cases when compared to healthy patients (16).

*F. prausnitzii* is the only known species of the genus Faecalibacterium, which belongs to the phyla Firmicutes. It is a key synthesiser of short-chain fatty acids (SCFAs), more specifically butyrate. There are three SCFAs that have anti-inflammatory properties and all are generated as a by-product of resistant starch fermentation: acetate, propionate and butyrate (17). A decreased amount of *F. prausnitzii *has been noted in patients with IBD, which is also known to secrete metabolites that impede nuclear factor-κappaB activation and IL-8 production, ultimately leading to anti-inflammatory effects (18,19). Furthermore, butyrate can thwart GI mucosa atrophy by being used as a nutrient for colonocytes, thus also hindering colonocyte autophagy (20, 21). Therefore, while some species of the GI microbiome can exacerbate inflammation, others are able to alleviate it.

The primary changes detected specifically in UC are: a decrease in diversity, stability, and numbers of certain bacterial species (7). The consequence of these changes, and the interactions with therapeutic interventions used in UC, for example, aminosalicylates, corticosteroids and immunosuppressive drugs are unknown. In one study, exploiting 16S rDNA based single strand conformation polymorphism (SSCP), as well as other molecular techniques in patients with active UC, displayed a reduced GI microbiome diversity (22). Conversely, a following study using similar procedures found members of the Firmicutes and Bacteroidetes phyla reduced in some cases of active UC, but not all (23). Investigating faecal samples, which were done by Willing *et al*. (24), amassed from a group of twins who were concordant or discordant for UC or CD respectively, showed no significant variances between healthy cases and IBD profiles. Interestingly, a twin study which analysed microbiota profiles using 16S ribosomal DNA (rDNA) sequences from intestinal biopsy samples, found UC patients had lessened bacterial diversity and greater Proteobacteria and Actinobacteria, with fewer Bacteroidetes in comparison with their healthy counterparts (25). Furthermore, healthy UC discordant siblings’ pairs showed a decreased bacterial diversity compared to healthy patients without a sibling with IBD, which indicates a possible inheritance pattern (25). Biodiversity of the dominant microbiota, which is decreased in UC, has been linked with temporal instability (26).

In CD, it has been found that intestinal inflammation, regardless of NOD2/CARD15 status, resulted in a decreased amount of commensal anaerobic bacteria (27). Using a metagenomic approach, Manichanh *et al* .(28) showed a marked reduction of diversity in patients with CD in comparison with healthy controls. This was due to the diminished complexity of Firmicutes, more specifically *Clostridium leptum* in patients with CD. As in UC, biodiversity of the dominant microbiota in CD has been linked with temporal instability (29). Willing *et al* (24) conducted a study looking at groups of twins who were concordant or discordant for UC or CD in which not only patients with CD had a different GI microbiome profile to their healthy counterparts, but also patients with intestinal inflammation primarily affecting the ileum, had a differing microbial profile to patients that had mucosal damage to the colon. Patients with CD in the ileum had a reduction of core bacteria, which include Faecalibacterium and Roseburia, and had augmented quantities of Enterobacteriaceae and *Ruminococcus gnavus*. These findings have been corroborated in multiple studies (13,19,24,28).

A decline in *F. prausnitzii *is associated with an increased risk of recurrent ileal CD postoperatively and has also been found in mouse models of intestinal inflammation that when given, has anti-inflammatory effects (19,30). Numbers of *F. prausnitzii *in active CD are much lower than in healthy patients and there are reduced numbers in patients with infectious colitis, hinting that a decrease of *F. prausnitzii *is secondary to intestinal inflammation or diarrhoea (13, 30). Enterobacteriaceae, a particular species of *E. coli*, are regularly found to be increased in CD patients, more so in faecal samples than the mucosa (31). A pathogenic group of *E. coli*, known as adherent-invasive *E. coli* (AIEC), has been found in sites of the ileal mucosa in patients with CD (32). AIEC was demonstrated to attach itself to intestinal epithelial cells, invade them and able to avoid phagocytosis and eradication by replicating at a high rate in phagolysosomes within macrophages (32). Interestingly, AIEC is not only present in healthy patients, albeit with a decreased quantity than in patients with CD, but also proven to not affix itself to intestinal epithelial cells in healthy patients (32). This result suggests that AIEC is explicitly concomitant with patients with CD in the ileum.

In summary, dysregulated immune responses and signalling with intestinal microbiota is heavily implicated in IBD. The deficit of tolerance with commensal microbiota in IBD patients is thought to be multifactorial with the vulnerability to specific genes, damage to the mucosal barrier and gut microbial dysbiosis all playing a significant role. This hypothesis is currently being researched thoroughly and will continue to be of focus in the future.

**Faecal microbiota transplantation in inflammatory bowel disease**

The first study utilizing FMT in the treatment of UC was in 1989, with a case series published the same year (33,34). Both reports identified remission of the disease despite the termination of immunosuppression techniques. Since these attempts, recent FMT trials in UC have reported remission rates between 0 to 100% (35-40). To date, there are two papers published as randomised controlled trials employing FMT as a therapeutic intervention in active UC (39,40). Moayyedi *et al*. (39) found that FMT induced a larger change in the GI microbiome than placebo, with a statistically significant remission in 24% of patients in comparison to 5% who achieved remission with a placebo. However, Rossen *et al*. (40) witnessed a statistically insignificant difference, where 30.4% of patients with UC achieved remission with FMT in comparison to 20.0% healthy controls. The contrasts between the two studies may be rationalised due to many differences in study design, placebo procedures, different definitions and time points of remission (table 1). Interestingly, Rossen *et al.* (40) used a suspension of autologous faeces as a control and two large stool weight (approximate 120g) FMT deliveries via nasoduodenal tube. The control could change the GI microbiome and lead to an increase in diversity as was witnessed in the placebo group, which can most likely be explained by a reduction in mucosal inflammation. Moayyedi *et al*. (39) used retention enemas to deliver 8.3g of stool per week of FMT. The lack of standardisation of application of FMT as well as stool preparation methods make it a challenge to ascertain which protocol is the most effective one. Further studies need to be undertaken to enhance the methods of FMT in UC. 

**Table 1:** Randomised controlled trials (RCT) published as a full paper utilising faecal microbiota transplantation (FMT) as a therapeutic intervention in Ulcerative Colitis (UC

Study	Number of Patients	Study Design	Primary Clinical Outcome	Technique of FMT application	Technique of placebo application	Remission patients, %	Response patients, %
Moayyedi et al. (39), 2015	75 (38 FMT, 37 placebo)	Double-blind RCT, placebo-controlled, randomized in a 1:1 ratio. Flexible sigmoidoscopies at baseline and week 7 post FMT	UC remission at week 7	Retention enema, 50mL from healthy donors, once a week for 6 weeks	Retention enema, 50 mL water, once a week for 6 weeks	FMT: 9/38 (24%) Placebo: 2/37 (5%)	FMT: 15/38 (39%) Placebo: 9/37 (24%)
Rossen et al. (40), 2015	48 (23 FMT, 25 control)	Double-blind RCT, placebo-controlled, randomized in a 1:1 ratio. Clinical and endoscopic follow-up was performed 6 and 12 weeks after first treatment	UC remission at week 12	Nasoduodenal tube, 500mL faecal suspension from healthy donors, 2 infusions 3 weeks apart	Nasoduodenal tube, 500mL suspension of autologous faeces, 2 infusions 3 weeks apart	FMT: 7/23 (30.4%) Control: 5/25 (20.0%)	FMT: 11/23 (47.8%) Control: 13/25 (52.0%)

There are several reasons accounting for the variation of effectiveness of FMT as a therapeutic intervention in UC. First, studies which used FMT recurrently over a select period of time (36,39) had greater remission rates than those which spread out the use of FMT (38,40). Moayyedi *et al*.(39) found that patients undergoing immunosuppressive therapy, and a briefer time suffering from UC, had a superior response to FMT intervention. Furthermore, patients receiving donated faecal samples did particularly well from one individual donor in comparison to the others, indicating that the composition of donated faecal sample could potentially be a factor in the success of treatment response. This notion can also be corroborated in another positive study, in which mixed enemas were generated from the use of 3-7 faecal donors (36), revealing that a combination of faecal donors may lead to increasing treatment success. The abundance of Clostridium clusters IV (which contains *F. prausnitzii)*, XIVa, and XVIII increased, while a reduction in Bacteroidetes was observed post FMT intervention in UC patients (40). These observations may be the key to re-establishing intestinal homeostasis via the effective detection and transplantation of explicit GI microbial communities. However, more work needs to be done to identify what constitutes a suitable faecal donor to use FMT as a treatment option in UC.

The use of FMT in CD has not yet been fully examined. There are several case series published in patients with CD (41-44). The outcomes of these reports demonstrate a mixed efficacy of the intervention on remission and response (table 2). *Cui et al*. (42) has the largest sample of patients with refractory CD, who underwent single FMT in the duodenum and had a clinical remission rate of 70% at 3 months. However, this decreased to 60% a further 3 months later (42). With CD being able to damage the GI tract, it is a more complex disease in comparison to UC. Taking this into account, and with the lack of data currently available to us, more work needs to be undertaken before FMTs efficacy in CD can be determined.

The majority of data regarding the safe use of FMT is available from studies in CDI (45). The safety profile of FMT in IBD is based on the few studies published, with the number of registered short term side effects found, being unexpectedly low. In a case series by Vermeire *et al.* (41), patients with administration of FMT via nasojejunal tube had more serious adverse effects (AE), including aspiration pneumonia. Hence, patients were then switched from FMT treatment through the upper GI tract to administration through a rectal tube (41). Rossen *et al*. (40) reported 78% of patients having FMT underwent mild AEs during or shortly after nasoduodenal tube insertion. The most common AE was transient borborygmus, followed by an intensification of stool regularity, with two patients vomiting after FMT infusion (40). In the same study, a patient was suspected to have a small bowel perforation five weeks post treatment; however, subsequently this was discovered to be severe small bowel CD (40). FMT applied via the upper GI tract has been linked with a high-grade fever more frequently than when utilising colonoscopy. However, Moayyedi *et al*. (39) who used a retention enema reported three patients (two in the FMT arc, one in placebo control) acquiring patchy inflammation of the colon and rectal abscess formation, with one FMT arc patient developing CDI after the study had finished. The former findings could be put down to the use of repeated enemas instead of an effect of FMT, as this AE was found in the placebo group too (39). With the latter finding, the faecal donor tested negative for *C. difficile*, thus making it unclear whether the FMT caused CDI in this patient. IBD flares in patients post FMT have also been reported; however, this was when FMT was utilised in CDI (46). If FMT can alter the GI microbiome with an aim to treat disease, it is also conceivable that transplantation could make things worse. We still do not fully know all of the pathogenic microorganisms present in faecal matter, and the unknown effect they may have on the recipient patient. These uncertainties highlight the paramount need for larger clinical trials not only focussing on the efficacy of the FMT, but also the safety profile.

**Table 2:** Case series published utilising faecal microbiota transplantation (FMT) as a therapeutic intervention in Crohn’s disease

Study	No patients	Efficacy endpoints	Technique of FMT application	Remission patients, %	Response patients, %
Vermeire et al. (41), 2016	6	Patients with endoscopic healing at week 8 post FMT	Nasojejunal tube & colonoscopy, 200 g stools homogenized with 400 mL, administered two times	0/6	0/6
Cui et al. (42), 2015	30	Disease activity assessed by Harvey-Bradshaw Index (HBI). Clinical improvement defined HBI > 3. Clinical remission defined HBI ≤ 4. Measured at 1 week, 1 month then at every 3 months post FMT till month 15	Gastroscopy into patient’s mid-gut (duodenum), 150-200 mL liquid suspension (~ 60cm^3^ faecal flora and ~ 100 mL saline) , single infusion	At 1 mo: 23/30 (76.7%) At 3 mo: 21/30 (70.0%) At 6 mo: 18/30 (60.0%)	At 1 mo: 26/30 (86.7%) At 3 mo: 24/30 (80.0%) At 6 mo: 20/30 (66.7%)
Vaughn et al. (43), 2014	8	Clinical remission defined HBI < 5 at week 4 and 8	Colonoscopy (ileum to rectum), 50g of stool suspended in 250ml of saline, single infusion	At 4 wk: 5/8 (63%) At 8 wk: 4/4 (100%)	N/A
Suskind et al. (44), 2015	9	Paediatric Crohn’s disease activity index (PCDAI) at 2, 6, and 12 weeks post FMT. PCDAI score of < 10 signifies remission, 10–29 mild disease, and ≥ 30 moderate to severe disease	Nasogastric tube, 30 grams of donor stool with 100–200 ml of saline, single infusion	At 2 wk: 7/9 (78%) At 6 wk: 5/9 (56%) At 12 wk: 5/9 (56%)	N/A

The treatment of rCDI in patients with IBD is yet to be fully evaluated. There are various studies by investigators, which all indicate that FMT does provide an appropriate substitute option in the therapeutic management of rCDI in IBD, albeit with a lower success rate (47-50). Patients with IBD present a significant challenge in both the diagnosis and management of CDI, with worse clinical outcomes and an increased mortality found (51). The safety profile of this intervention has also come into question with a recent study indicating that the rate of flare-ups of IBD increased by a quarter (48).

**Summary and future directions**

Studies in humans have authenticated the role of intestinal microbiota in the pathogenesis of IBD. FMT has been hypothesised as the best method to restore gut microbial dysbiosis to halt clinical disease progression. However, the efficacy of FMT as a therapeutic intervention in IBD continues to be unclear. 

With the current data published, we know that FMT does not have a similar influence on IBD in comparison to its effects on recurrent CDI. Both IBD and CDI are known modulators of the GI microbiome, however, IBD multifactorial and much more complex. Regarding UC, mounting evidence is being published proving that FMT has the potential to be effective in the management of IBD. For CD, more work needs to be done as it is unclear whether FMT is useful. Furthermore, there is no unanimity concerning the process of FMT. Variations of the procedure include, but are not limited to: multiple methods of selection and screening of faecal donors, the optimal technique of FMT application, comprising of the best volume, administration route, pre-treatment regimen, preparation before FMT, and short- and long-term efficacy and safety profiles. Additionally, the theoretical application of FMT is to restore the tampered homeostasis of the intestinal microbiota in IBD. To this effect, there are limited studies investing this hypothesis; thus currently the use of FMT is lacking evidential support. Future trials should illuminate the clinical use of FMT and its effect on manipulating the GI microbiome. A novel oral method of FMT application should also be established, such as microbiota-based pills which are safe and standardised. Thus in the future, an effective placebo could also be generated, allowing long-term AEs to be fully explored. 

FMT is a therapeutic intervention that is developing rapidly.  With the advent of personalised medicine, the future of GI microbiome profiling and providing tailored therapy is an exciting prospect. However, there persists to be many fundamental questions that have yet to be answered; resolving these issues are paramount to allow FMT to achieve a more prominent role in the management of IBD. 

## Conflict of interests

The authors declare that they have no conflict of interest.
